# Mature results from EORTC 22033: Implications of inferior results with monotherapy for grade 2 gliomas

**DOI:** 10.1093/neuonc/noag162

**Published:** 2026-07-20

**Authors:** Connor J Kinslow, David Schiff

**Affiliations:** Department of Radiation Oncology, Memorial Sloan Kettering Cancer Center, New York, New York, USA; Division of Neuro-Oncology, University of Virginia School of Medicine, Charlottesville, Virginia, USA

Several factors converged in the early 2000s to generate interest in a trial comparing chemotherapy to radiotherapy in low-grade gliomas. Among these was the emerging appreciation that these tumors, particularly oligodendrogliomas, were sometimes chemosensitive. Another was the development of a well-tolerated oral agent, temozolomide, recently approved for recurrent anaplastic gliomas. Lastly was the longstanding desire to defer radiotherapy when possible to mitigate potential delayed cognitive neurotoxicity in patients with relatively slow-growing tumors.

The culmination was EORTC 22033-26022, a phase 3 trial randomizing patients with low-grade gliomas to standard radiotherapy (50.4 Gy) versus 12 cycles of dose-dense temozolomide. Study inception antedated the discoveries of the presence of IDH mutations in low-grade gliomas as well as the benefits of combined chemoradiotherapy in glioblastoma and lower-grade gliomas. Eligibility required either uncontrolled seizures, neurological deficits, or age > 40 to exclude patients in whom observation was an option. Stratification included 1p deletional status to balance the arms for oligodendrogliomas. The primary endpoint was progression-free survival (PFS), with overall survival (OS) an important secondary endpoint. With 478 subjects and a median follow-up of 13 years, this is the largest clinical trial to date in low-grade gliomas.

Final results of EORTC 22033 have just been published.^1^ In short, the study found no difference in PFS and OS between the temozolomide and radiotherapy arms. OS among IDHmt astrocytomas was 6.6 years with radiotherapy and 6.7 years with temozolomide; the corresponding figures for oligodendrogliomas were 12.9 and 14.9 years. PFS, as previously reported, showed a trend favoring radiotherapy among IDHmt astrocytomas. As approximately 90% of progressing patients in each arm received second-line treatment, with two-thirds receiving the opposite arm as salvage, the results suggest OS with sequential monotherapy does not depend on sequencing. Curiously, among the 64 patients with IDHwt tumors, OS favored temozolomide. As in the recently published CATNON study of grade 3 astrocytomas, age >40 was not prognostically unfavorable when analysis was restricted to IDHmt tumors.

Results from two randomized clinical trials of chemotherapy versus radiotherapy (EORTC 22033, NOA-04) and four rando­mized trials of chemoradiotherapy versus radiotherapy alone (RTOG 9802, ECOG-ACRIN E3F05, RTOG 9402, EORTC 26951) have now demonstrated roughly equivalent outcomes with chemotherapy versus radiation monotherapy and the superiority of chemoradiotherapy for grade 2 or 3 gliomas. It should be noted that the relative (HR ∼0.20-50) and absolute magnitude of benefit (∼7 to >10 years) on OS with combined chemoradiotherapy is rare to encounter in oncology trials. The futility of either approach as a single-modality treatment may be explained in the context of other solid tumors. The modest doses of radiotherapy used in grade 2 glioma trials (50-54 Gy) are not definitive doses for other solid tumors in the absence of radiosensitizing chemotherapy. Higher doses (66-80 Gy) are often required to eradicate gross tumor when used as monotherapy. Single-agent chemotherapy also rarely provides local control of solid tumors and is generally used as a radiosensitizer. Although RTOG 9802 and EORTC 22033 used different definitions of “high-risk” low-grade glioma, across-trial ­comparisons yield remarkably similar PFS and OS times for the monotherapy arms (Figure 1).^1^^,^^2^ The 10-year PFS rates for IDH-mutant oligodendroglioma treated with chemoradiotherapy, chemotherapy, radiotherapy (RTOG 9802), and radiotherapy (EORTC 22033) were 83%, 24%, 21%, and 21%, respectively, and for IDH-mutant astrocytoma were 52%, 7%, 9%, and 16%. The OS rates for IDH-mutant oligodendroglioma were 94%, 64%, 72%, and 58% and for IDH-mutant astrocytoma were 62%, 28%, 23% and 32%, respectively. Also striking is that the outcomes for grade 2 IDH-mutant astrocytoma treated with radiotherapy in EORTC 22033 were similar to that of grade 3 IDH-mutant astrocytoma in the CATNON trial treated without adjuvant TMZ (PFS: 4.0 and 3.4 years, respectively; OS: 6.6 and 6.0 years), and approximately half that of the combined radiation/adjuvant TMZ arm on CATNON (PFS: 7.0 years; OS: 12.5 years).^3^ Taken together, these data strongly suggest that OS is compromised when chemoradiotherapy is not employed as the definitive upfront treatment strategy. While radiotherapy was historically delivered as a single course of treatment, chemotherapy regimens can be repeated during subsequent courses after tumor progression. There are several possible explanations for the inferiority of either approach when used alone:

**Figure 1.**
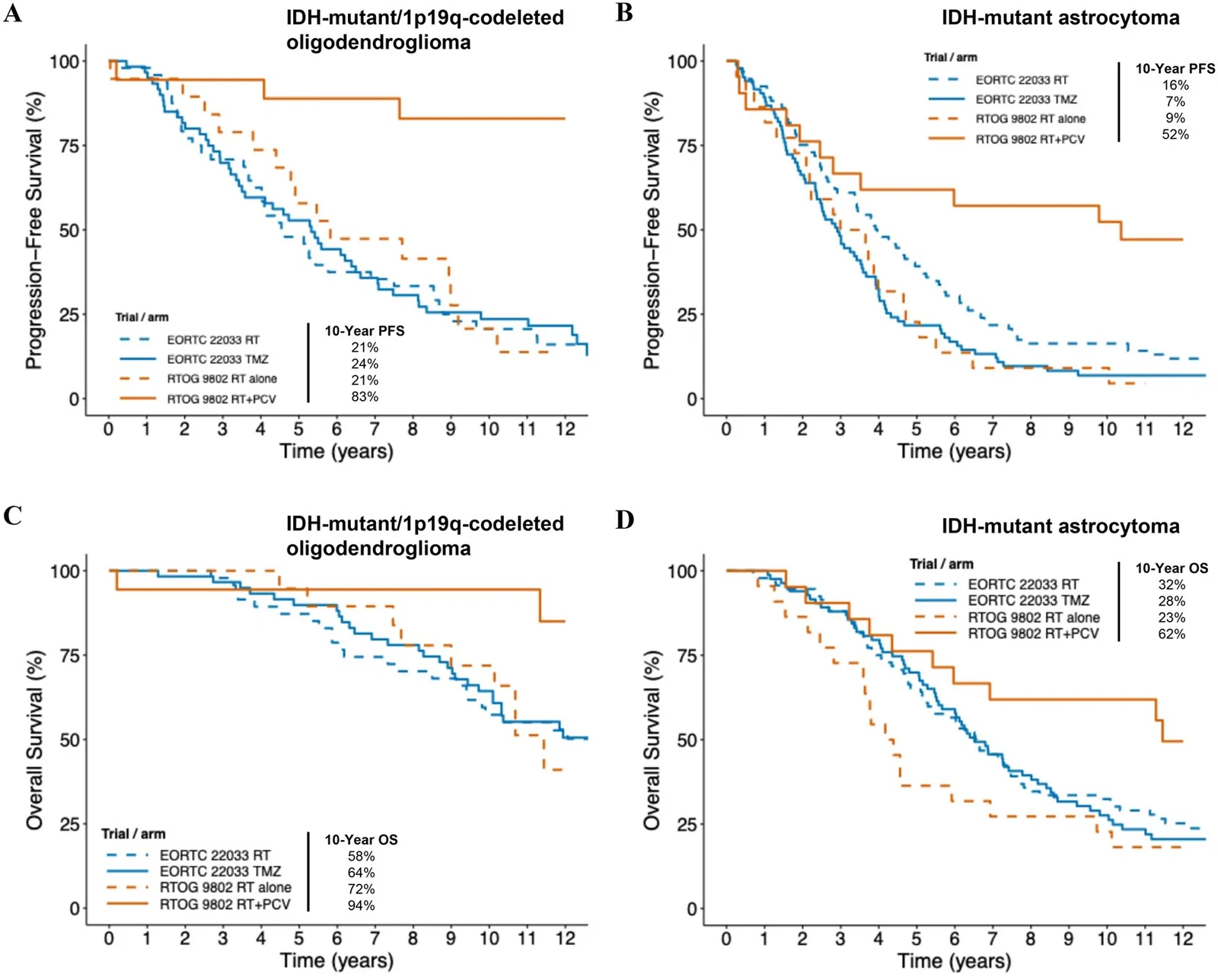
Superimposed Kaplan-Meier curves for progression-free survival (A-B) overall survival (C-D) from the EORTC 22033 and RTOG 9802 trials of high-risk low grade glioma, stratified by IDH-mutant and 1p19q-codeleted oligodendroglioma (A, C) and IDH-mutant astrocytoma (B, D). Individual patient-level data were estimated using WebPlotDigitizer 5.2 (Automeris LLC, California, USA). PFS- Progression-free survival; OS- Overall survival

1. Long-term survival is dependent on optimizing early tumor control. Delaying radiotherapy compromises early tumor control, rendering it less effective when delivered to larger tumor volumes as a salvage treatment.
2. Temozolomide induces acquired treatment-resistance, rendering tumors less sensitive to additional treatments at progression.
3. Patients in 22033 were generally treated with chemotherapy or radiation monotherapy at the time of tumor progression and, therefore, also received suboptimal salvage treatments.

Early results from EORTC 22033 further challenged the assumption that delaying radiotherapy would improve health-related quality of life (HRQOL) and neurocognitive preservation. Although patients in both arms reported deficits at baseline, patient-reported HRQOL and mini-mental status exams improved over time and were not different between arms at 3 years.^4^ However, limited compliance with questionnaires beyond 3 years precluded analysis at later timepoints. Progression of disease, rather than receipt of radiotherapy or radiation dosimetry, was the dominant cause of clinical decline at early timepoints.^4-7^ While older non-randomized studies of patients treated predominantly in the 1970s and 1980s with now outdated techniques (whole brain radiotherapy, 2-dimension radiotherapy, >2.2 Gy/fraction, >60 Gy) ­suggest late radiation-related neurocognitive decline,^8^ the majority of modern series have reported stable long-term neurocognition with modern IMRT/VMAT techniques that use improved beam modulation and limit hotspots.^9^ Still, these small non-randomized comparisons measuring group averages may fail to detect a subset of patients who develop clinically significant neurocognitive decline after IMRT. Therefore, the issue remains open to debate. The ongoing NRG-BN005 trial, which randomizes patients with grade 2-3 IDH-mutant glioma to proton or photon radiotherapy, will provide valuable long-term data using robust neurocognitive primary endpoints but will be limited to patients who receive radiotherapy.

Applying these data to the current INDIGO era,^10^ where vorasidenib is now the predominant strategy to delay definitive treatment, similar questions remain: (1) Will the modest tumor control demonstrated with vorasidenib, when compared to historical trials of chemoradiotherapy, lead to earlier tumor progression and death? (2) Does vorasidenib induce mutational or epigenetic changes that lead to cross-resistance or poorer efficacy of chemoradiotherapy as a salvage treatment? Or, on the contrary, will having an additional tool in the grade 2 glioma arsenal with an orthogonal mechanism extend OS by increasing the number of viable salvage options? (3) Will the use of vorasidenib in the upfront setting preserve quality of life and neurocognition, or will tumor progression be the dominant competing cause of clinical and cognitive decline? As there are currently no trials that compare vorasidenib to chemoradiotherapy, there are no direct pathways to answer these questions. Additional combination strategies, such as vorasidenib with chemoradiotherapy, immunotherapy, or other targeted therapies, warrant further investigation. Perhaps we will see improved OS with “vora- chemoradiotherapy” over chemoradiotherapy alone! The landscape of grade 2 gliomas has shifted over the past 25 years, but how we define “high-risk” and how these definitions influence the optimal timing of chemoradiotherapy remain relevant.
